# Cancer survivorship and functional health: what we need to address in an aging cancer population

**DOI:** 10.2340/1651-226X.2025.43076

**Published:** 2025-03-19

**Authors:** Anja Mehnert-Theuerkauf, Susanne Oksbjerg Dalton, Christoffer Johansen

**Affiliations:** a Department of Medical Psychology and Medical Sociology, Comprehensive Cancer Center Central Germany (CCCG), University Medical Center Leipzig, Leipzig, Germany; b Cancer Survivorship, Danish Cancer Institute, Copenhagen, Denmark; c Faculty of Health Sciences, Institute of Clinical Medicine, Copenhagen University, Copenhagen, Denmark; d Department of Clinical Oncology and Palliative Care, Zealand University Hospital, Næstved, Denmark; e Department of Oncology, CASTLE, Copenhagen University Hospital, Copenhagen, Denmark

Over the past decades, the global burden of disease has shifted from communicable to non-communicable diseases (NCDs) and from premature death to years of life with disability [1]. NCDs, however, are responsible for 41 million deaths per year globally, accounting for 74% of all deaths worldwide. Together with cardiovascular diseases, chronic respiratory diseases and diabetes, cancer is one of the four prevalent diseases responsible for over 80% of all premature NCD deaths [1].

The good news is that numerous studies now show relatively consistently that about 40% of cancers as well as other NCDs could be prevented [2, 3]. However, this number is also disappointing from a public health perspective: Risk modification has played a relatively minor role in recent years, although it has great potential to improve the health of both individuals and society as a whole [4, 5]. As often described, tobacco and harmful alcohol consumption, physical inactivity, unhealthy diet and metabolic risks as well as infections and air pollution increase the risk of dying from cancer – risks, some of which could certainly be modified [6, 7].

These findings are also highly relevant to cancer survivorship. In addition to biological factors, the risk of disease progression, recurrence or second primary cancers is also associated with lifestyle habits [8, 9]. Consequently, adopting a healthy lifestyle is imperative in order to prevent and alleviate the occurrence of serious biopsychosocial late- and long-term consequences [7, 10, 11]. For instance, a cancer-registry based study of depression and anxiety in long-term hematologic cancer survivors demonstrated a significantly elevated relative risk for anxiety and depression, persisting for more than 12 years following diagnosis, in comparison to the age- and gender-matched comparison sample from the general population. Patients in middle age, around 65 years, were particularly affected [12]. The detrimental effects of depression on health [13] are significant, including its considerable impact on quality of life and the development of secondary conditions, such as cardiovascular late effects, in addition to chemo-radio toxicities. These factors emphasise the importance of early detection and treatment of psychosocial distress [14–16], as well as the implementation of healthy lifestyle changes [17, 18].

Nevertheless, evidence shows that despite the potential of a cancer diagnosis to encourage people to adopt a healthier lifestyle, a significant proportion of cancer survivors are physically inactive and overweight. Only a small proportion between 7 and 40% follow the recommendations for a healthy lifestyle [19, 20]. Lower health literacy is one of the main underlying causes associated with lower socioeconomic status, high comorbidities, lack of resources and barriers to timely access to health services and effective treatments [21–24].

The importance of strengthening health literacy and tertiary prevention in the context of cancer survivorship is particularly plausible in view of the fact that we are dealing with an increasingly large and ageing cancer population that not only suffers from cancer, but also from a high level of comorbidity overall [25, 26]. The title chosen by the authors Bluethmann et al. [27] for their paper ‘anticipating the silver tsunami’, published almost 10 years ago, may have been somewhat gimmicky, but it also gets to the heart of the problem. According to the prognoses, the majority of cancer survivors will be over 75 years old in 2040 [27, 28].

The European Cancer Survivorship and Rehabilitation Symposium (ECRS) 2024 in Copenhagen demonstrated the extensive nature of the biopsychosocial late- and long-term problems, which are closely interlinked. Thematic areas of focus encompassed novel immunotherapies for haematological malignancies, mental health challenges, lymphoedema, sexual dysfunction, survival after metastatic cancer and enhancing the quality of care for survivors of rare cancers.

A significant theme of the conference was research into the long-term and late effects of cancer therapies, which have been shown to limit the quality of life of patients both with cancer-free survivals and those with metastasised cancer. Larsen et al. [29] emphasise the need for a broader approach to the treatment of lymphoedema in different cancer diagnoses, as lymphoedema symptoms are common among cancer survivors and correlate with more depression and pain disorders as well as lower health-related quality of life (HRQoL). The findings by Pappot et al. [30] demonstrated that increased toxicity across all lines of treatment contributes to an impaired quality of life, thus emphasising the necessity of treating both acute and chronic symptoms in the care of metastatic breast cancer survivors. Clear communication about prognosis is vital for informed decision-making and aligning care with patients’ life goals. New care models should blend survivorship and palliative care, focusing on equitable access to tailored services, considering recent treatment and prognosis changes in patients with advanced or metastatic cancer such as the care model presented by Lai-Kwon et al. [31].

Langballe et al. [32] address the issue of long-term distress and the usefulness of distress screening in the context of breast cancer survivorship. With regard to cancer surveillance in the survivorship phase, Lindahl et al. [33] emphasise a risk-adapted surveillance approach based on identified risk factors for relapse in seminoma survivors. Stegenborg et al. [34] deal with socioeconomic disparities and childhood cancer survival, particularly with regard to children from disadvantaged families.

Thyø et al. [35] and Nahavandipour et al. [36] looked at sexual dysfunction and sexual stress in cancer survivors and showed that almost half of sexually active men with rectal cancer resigned from their sex life within a year due to erection problems [35] and that the prevalence of sexual stress and impairment is high in men with different types of cancer [36]. Both studies underscore the fact that healthcare professionals should pay more attention to sexual health in all men with cancer.

A number of articles examined the effectiveness of interventions for various symptoms and problems in cancer survivors, such as quality of life, work ability or sleep. These interventions included the Guided Self-Determination (GSD) programme in the MyHealth study [37, 38] and the SleepNow intervention [39].

However, despite our growing understanding of the variety of survivorship challenges, it is evident that we are only at the beginning of actually establishing and implementing cancer survivorship care plans that are grounded in empirical evidence and demonstrate tangible effectiveness [40]. In the context of health services and implementation research, the existence of effective interventions and programmes is not the only relevant consideration. Equally important is research into effective ways of implementing these programmes in everyday care, with a view to ensuring optimal target group care.

The demographic shift towards an ageing population combined with a lack of health literacy poses a major challenge for health policy and for us as a society. Achieving good functional health after a cancer diagnosis is therefore an important goal of cancer survivorship research for the coming years. Functional capacity plays a central role in shaping the health of the population as a whole and the well-being of society, emphasising the importance of health both for the individual and for society as a whole [41]. Functional health has recently been posited as a third health indicator alongside morbidity and mortality [41]. In the new model, functional health comprises two aspects: biological health, which refers to the physiological and psychological functions and anatomical structures of the body that represent an individual’s intrinsic ability to perform human activities; and lived health, which refers to the actual performance of activities in interaction with the physical, built and social environment.

In order to achieve better biopsychosocial outcomes and enhanced functional health following a cancer diagnosis, further research is required to address the following dimensions [42, 43]: Firstly, the enhancement of (tertiary) prevention and the implementation of a healthier lifestyle, particularly among high-risk groups, should be prioritised. This encompasses the cessation of tobacco and alcohol consumption, the adoption of a healthy diet and maintenance of optimal body weight, the engagement in regular exercise, the utilisation of sun protection measures, the mitigation of exposure to air pollution, and the completion of human papillomavirus vaccinations. Secondly, the reinforcement of health literacy, the facilitation of early detection and screening measures, and the enhancement of treatment and comprehensive care are imperative. This encompasses the collaborative management of treatment and health goals, and the facilitation of access to supportive services that promote physical and psychosocial wellbeing.
